# TIPP-SD: A new method for species detection in microbiomes

**DOI:** 10.1371/journal.pcbi.1014347

**Published:** 2026-05-28

**Authors:** Chengze Shen, Eleanor Wedell, Mihai Pop, Tandy Warnow

**Affiliations:** 1 Siebel School of Computing and Data Science, University of Illinois Urbana-Champaign, Urbana, Illinois, United States of America; 2 Department of Computer Science, University of Maryland at College Park, College Park, Maryland, United States of America; Fudan University, CHINA

## Abstract

In this study, we present TIPP-SD (i.e., TIPP for Species Detection), a new technique for species detection in a microbiome sample. TIPP-SD uses a substantially modified version of TIPP3, which is a recently developed abundance profiling tool based on maximum likelihood phylogenetic placement into marker gene taxonomies. TIPP-SD depends on a parameter (i.e., “threshold”) for the required support for species detection, thus allowing us to compute a precision-recall curve as we vary this parameter. In comparing the precision-recall curves for TIPP-SD, TIPP3, Kraken2, Bracken, Metabuli, and Metapresence, we find that TIPP-SD improves on the other methods with respect to accuracy under conditions where there is a highly variable distribution of species abundance or where there is sequencing error. Under other conditions, TIPP-SD is close to the best of these methods. Finally, although TIPP-SD is slower than the other methods, it is still fast enough to be used on large datasets. TIPP-SD is available in github as part of the TIPP3 software package.

## Author summary

We present TIPP-SD, a new method for species detection in microbiome samples. Like its predecessor TIPP3 (PloS Computational Biology 2025), TIPP-SD uses maximum likelihood phylogenetic placement into taxonomies based on marker genes. However, to provide high accuracy for species identification, TIPP-SD uses a novel way of combining statistical support obtained from these placements. Our experiments on a wide range of datasets show that TIPP-SD provides improved accuracy compared to well-established methods, such as Kraken2, Bracken, Metabuli, and Metapresence, with the greatest improvements when the species abundance is highly variable or the sequencing technology introduces high error rates.

## Introduction

Abundance profiling and species detection are two related problems in microbiome analysis. Species detection aims to list the species found in an environmental sample, while abundance profiling aims to estimate the distribution of species (or genera, families, etc.) within the sample. Each of these problems has broad applications and together they provide insight into questions related to ecology, evolution, and human health [1–5].

Some of the methods for these problems include Bracken [6], Kraken2 [7], krepp [8], MEGAN4 [9], Metabuli [10], MetaPhlan4 [11], Metaphyler [12], mOTU [13], sylph [14], the TIPP family of methods [15–17], and YACHT [18]. While methods for abundance profiling can be used for species detection by setting a threshold above which a species is considered to be detected, the selection of the appropriate threshold is itself a non-trivial question. In addition, because the goal of abundance profiling is an estimate of the distribution (relative frequencies) at a given taxonomic level, such as species, abundance profiling methods can afford to ignore very low abundance taxa, as setting their frequency to zero is unlikely to hurt the overall accuracy by more than a very small amount. However, such a strategy can reduce their utility for species detection. Other issues in using abundance profiling for species detection are discussed in [19], who specifically emphasize the importance of detecting species that are present in very low frequencies (i.e., “rare taxa”), and argue that abundance profiling methods are insufficient for this purpose. For multiple reasons, therefore, methods specifically designed for species detection are of interest.

In this study, we propose TIPP-SD, a new technique for species detection. While TIPP-SD builds on the basic algorithmic design in TIPP3 [17], in order to achieve high precision and recall for species detection, it modifies the TIPP3 technique in several ways.

We compare TIPP-SD to the naive use of TIPP3, and show that TIPP-SD provides superior accuracy for species detection. We also compare TIPP-SD to Bracken, Kraken2, Metabuli, and Metapresence [19]. We find that TIPP-SD has very good accuracy, with improved accuracy compared to the other methods when species abundance is variable or when there is a high rate of sequencing error. We also find that TIPP-SD is slower than Kraken2 and Bracken, but is still fast enough to be used on very large datasets. The TIPP-SD software is available in open-source form on github as part of the TIPP3 package [20].

## Background

We provide some background here, including a description of the TIPP3 method for abundance profiling.

### Abundance profiling methods based on marker genes

The goal of abundance profiling is to obtain a good estimate of the distribution of the species, genera, families, etc., in a given microbiome sample. While this problem can be considered at different taxonomic levels, for the purpose of this discussion, we will assume the interest is in estimating the abundance at the species level.

The input is typically a set of reads, though in some cases contigs may also be used, which could be based on amplicon sequencing (e.g., just one gene) or metagenomics (from across the genome). Because the goal is to produce a good estimate of the relative frequencies of species, abundance profiling methods must consider factors that distort the estimates of relative abundance: the genome length and whether genes appear in multiple locations within a genome [21,22]. Because of these challenges, *some* methods for abundance profiling are based on filtering the input reads to those that are derived from *marker genes*, which are genes that are expected to be single-copy and universal. Examples of such methods, which are referred to as “marker gene-based methods,” include Metaphyler [12], MetaPhlan [23], mOTUs [13], and the TIPP family of methods [15–17]. Of these, TIPP3 [17] has been shown to have the best accuracy for abundance profiling, especially when working with reads generated by sequencing technologies that have high error rates (indels or substitutions), such as nanopore [24] and PacBio [25]. As shown in [17], restricting Kraken2, Bracken, and Metabuli to the reads that are mapped to the TIPP3 marker genes *improved* the abundance profiling distributions that each method produced. Furthermore, even after this modification to Kraken2, Bracken, and Metabuli, TIPP3 was still more accurate than Kraken2, Bracken, and Metabuli in nearly all conditions; the exceptions were limited generally to Illumina reads (which have very low error rates). TIPP3 was also more accurate than Metaphlan4 [11], which is another marker-gene based method.

Because TIPP3 had high accuracy for abundance profiling, we seek in this study to build on its technical approach, but modify it so as to obtain high accuracy for the species detection problem.

### TIPP3

We begin with a description of the high-level approach of the TIPP3 method; see [17] for full details. The (publicly available) TIPP3 reference package includes multiple sequence alignments and taxonomies for each marker gene in the package. TIPP3 uses BLASTN [26] to map reads to the TIPP3 marker gene reference package. Any read that does not map to a marker gene is discarded; this is referred to as “filtering.” Each read that maps to a marker gene is added into the corresponding marker gene alignment using WITCH [27].

The next step in the analysis places each read into the taxonomy for that marker gene, using a phylogenetic placement method based on maximum likelihood, such as pplacer [28], EPA-ng [29], or BSCAMPP [30] (a divide-and-conquer approach that enables pplacer and EPA-ng to be used on large trees). Of these phylogenetic placement methods, pplacer is the most accurate but cannot be used on large trees very easily. EPA-ng is faster than pplacer and slightly less accurate. By default, TIPP3 uses pplacer-taxtastic [31,32], a way of using pplacer that allows it to run on moderately large trees.

The output from the phylogenetic placement step is a list of the top edges in the tree (using maximum likelihood as the criterion), along with the relative support for each edge. TIPP3 picks the edge in the rooted taxonomy so that the support for the read belonging to the clade below that edge is at least *B*, where *B* is an input parameter set to 0.95 or 0.9 depending on whether EPA-ng or pplacer is used for phylogenetic placement. The selected edge determines the taxonomic labels for the read: it may be given a species label, or it may only be placed at a genus or higher level. Finally, the taxonomic information across all the reads that map to marker genes is aggregated to form an abundance profile at each taxonomic level.

A fast version of TIPP3 (TIPP3-fast) makes two adjustments to the TIPP3 design in order to reduce the runtime, with a small increase in error: instead of using WITCH to add reads into the gene alignment it uses BLASTN, and instead of using pplacer-taxtastic for phylogenetic placement it uses BSCAMPP with EPA-ng.

## TIPP-SD

We now describe TIPP-SD. We describe it here for species detection, but note that the algorithm can be generalized to detect other taxonomic levels as well.

TIPP-SD has many similarities to TIPP3, but makes specific changes that improve its accuracy for species detection. TIPP-SD uses the same reference package as TIPP3 of multiple sequence alignments and taxonomic trees for each of its marker genes. Like TIPP3, TIPP-SD filters the reads to keep only those that map to its reference set of marker genes, and then processes each read in a similar fashion as TIPP3 (i.e., using a read alignment step followed by a phylogenetic placement step), with choices for each of these steps. Recall that given a single read that is mapped to the marker gene *g*, each phylogenetic placement method returns support values for the top edge placements in the tree, based on their likelihood values. If none of the top edges are at the species level, then the read will not be classified at the species level. After this is completed, TIPP-SD decides, for each species, whether it is present in the input, based on a user-provided support threshold. This last step is achieved using one of two techniques that we explored: *marker vote* or *marker confidence*. It is this last step that distinguishes TIPP-SD from TIPP3. Because of this, we describe the marker vote and marker confidence techniques in detail.

Recall that only some reads map to marker genes, and of these some will not be eligible for species classification if the phylogenetic placement method does not find any edge (defining a species) to have high support. For those reads that are eligible for species classification, the two methods operate as follows.

### Marker vote

Each read that is eligible for species classification is assigned the species that has the highest support from the phylogenetic placement method. Each such read has already been mapped to a marker gene, and we consider the marker gene to have therefore voted for that species. Thus, after aggregating results from all mapped reads, we will have a collection of species, each with a list of marker genes that voted for the species. We let *vote*(*s*) denote the number of votes for the species *s*.

In the TIPP3 reference package, a species may not appear in all the marker gene trees, depending on the quality and completeness of the genomes for the species. Therefore, for each species *s*, we note the number *m*_*s*_ of marker genes in which *s* appears. The ratio $\frac{vote(s)}{m_{s}}\in [0,1]$ then represents the fraction of marker genes that vote for the presence of species *s*. By setting a threshold *T* and checking if $\frac{vote(s)}{m_{s}}\geq T$, we can determine if a species *s* is detected. Thus, the output of marker vote depends on the threshold *T*.

### Marker confidence

Here we describe a technique for assigning a confidence for a species being present that takes into account the statistical support values for a read to belong to each of the species, as provided by the phylogenetic placement method during the read placement stage. Thus, we do not select the single best species assignment for a read, but consider all the assignments with positive support, and then normalize appropriately. We describe this technique in detail below.

For a marker gene *g* and a species *s*, we collect all reads that are assigned to *g* and have non-zero placement supports for *s*, and we take the placement support average. This is considered the *confidence score* for species *s* from marker gene *s*, denoted as *c*(*g*, *s*). Let *G*_*s*_ be the set of marker genes that species *s* has in the TIPP-SD reference package. Thus, we can compute the “marker confidence” score of species *s* supported by marker genes *G*_*s*_ as:


$$C_{s}=\frac{\sum_{g\in G_{s}}c(g,s)}{\left|G_{s}\right|}$$

By setting a threshold *T* on marker confidence, TIPP-SD can effectively determine which species is confidently detected in the input sample, if *C*_*s*_ ≥ *T*.

## Experiment design

In our experiments, we explore different methods for identifying species on datasets generated with varying types of sequencing technologies, where we know the true list of species present in the sample. This allows us to quantify both precision and recall for each analysis, and to explore the conditions under which each method is accurate.

### Benchmark datasets

A summary of the datasets we used and their properties can be found in Table 1. We provide more details for the read simulation and each dataset in the following paragraphs. The simulated reads generated for training data are available in the Illinois databank [33]; the scripts for generating the testing data are available on github at [20].

**Table 1:** Properties of simulated datasets. The 50 known genomes were used for designing TIPP-SD and the other genomes were used for evaluating TIPP-SD in comparison to other methods.

Dataset	Type	# species	# reads	Avg length	Avg cov
50 known (TIPP-SD design)	Illumina	50	10,500,910	150	20
	PacBio	50	1,047,884	3006	20
	Nanopore	50	184,327	4033	5
1000 known (testing)	Illumina	1000	13,795,579	150	1
	PacBio	1000	1,418,197	2925	1
	Nanopore	1000	921,633	4036	1
	Nanopore	1000	9,216,327	4032	10
CAMI II marine (testing)	Illumina	301	33,301,262	150	2
	PacBio	301	1,641,591	2968	2

#### Sequencing models.

We used read simulators to generate Illumina, PacBio, and nanopore reads of each given set of genomes, as described below (except for CAMI II datasets, which are available online). We used art_illumina (v2.5.8) [34] for generating Illumina short reads with the HS25 error model (fixed 150 bp in read length, ∼0.1% substitution rates). We used PBSIM [35] for generating PacBio long reads with the CLR sequencing error model, with an average read length of 3000 bp and a minimum length of 400 bp to understand the impact of sequencing error. The CLR model has an average 78% sequencing accuracy, with 3.23% substitution rate, 10.53% insertion rate, and 3.98% deletion rate (obtained by aligning simulated reads to reference using LAST [36]), We used Nanosim (v3.2.2) [37] with a pretrained metagenome model of bacteria community to simulate nanopore reads with a total error rate of 11.3% (3.9% substitution, 3.2% insertion, and 4.2% deletion rates).

#### 50 known genomes.

We used the same known genome datasets from the TIPP3 study [17], with 50 genomes that correspond to 50 different species, in Illumina, PacBio, and nanopore reads. “Known” means that these genomes are present in the reference package for TIPP-SD. These datasets have a coverage of 20 for all included genomes except for nanopore reads, which have a coverage of 5. Note that the 50 species have the same abundance level. This dataset was used in Experiment 1, where we designed TIPP-SD.

#### 1000 known genomes.

We also generated a new dataset with 1000 genomes that correspond to 1000 different species. Like the 50 known genomes dataset, these are referred to as “known” because they are drawn from the TIPP-SD database. We set the coverage of read simulations to 1 to imitate low species presence in a microbiome sample. We also generated a separate set of nanopore reads with a coverage of 10 to study the impact of higher coverage on the methods’ performance in recall and/or precision of species detection. Note that the 1000 species have exactly the same abundance; thus, each species is expected to appear in 0.1% of the sample. This dataset was used in Experiment 2, where we compared TIPP-SD to TIPP3, Bracken, Kraken2, and Metabuli, and in Experiment 3, where we compared TIPP-SD to Metapresence.

#### CAMI II marine dataset.

We included the Illumina and PacBio reads from the CAMI II marine dataset, replicate 1 [38]. These two datasets were also studied in [17]. Replicate 1 contains 301 species with non-zero estimated abundance, which are considered for species detection. This dataset has a wide range of estimated abundance levels, ranging from well under 0.001% to more than 10%. The estimated species abundances are computed using the abundance value inputs to the simulator, normalized by the total abundance, and so are *estimates* of the actual abundance in the simulated data. Hence, some species with very low estimated abundance may not even contribute reads in the simulated dataset. This dataset was used in Experiment 2, where we compared TIPP-SD to Bracken, Kraken2, and Metabuli.

### Methods

We compared TIPP-SD to TIPP3 (v0.3), Kraken2 (v2.17.1), Bracken (v3.0.1), Metabuli (v1.1.1), and Metapresence (v1.0). Within a given experiment, the methods that are compared have databases that contain exactly the same set of genomes, thus ensuring that the comparison is fair. While TIPP-SD is always run with filtering (i.e., removing reads that do not map to its marker genes), this is optional for the other methods. Here, we briefly describe each method and the databases they use in comparison to TIPP-SD; for additional details, version numbers, and commands, see S1 Appendix.

#### TIPP-SD.

TIPP-SD has several algorithmic parameters: the read alignment method, phylogenetic placement method, and choice between Marker Vote or Marker Confidence (see Table A in S1 Appendix). In Experiment 1, we set defaults for these parameters using the Algorithm Design datasets (i.e., the 50 known genomes datasets), and then used the default setting in subsequent experiments.

#### Kraken2 and Bracken.

Kraken2 and Bracken are both taxonomic identification tools that have been used for abundance profiling. CAMI II [38] includes Kraken2 and Bracken and shows that each can be used for species detection based on the number of reads that are assigned (in some way) to a species, and has examined this question based on just having a single read assigned. Other studies also use Kraken2 and Bracken for species detection based on the number of reads assigned to a species (e.g., [18,39]) or use Bracken for species detection based on the confidence score reported by Kraken2 [40].

Kraken2 uses its database and does k-mer searches for read classification. Using the genomes from the TIPP-SD reference package, we built custom databases for Kraken2 and Bracken. For our Illumina style reads, we use the default settings, and for PacBio and nanopore style reads, we use a k-mer size of 26 as recommended by [41].

Bracken uses the Kraken2 report directly as input, as well as a database generated from the same genomes as Kraken2 but with different k-mer and read length settings (detailed in Sect A in S1 Appendix).

#### Metabuli.

Metabuli is a method for abundance profiling and metagenomic classification. It uses structures similar to k-mers called metamers in order to classify reads and then reports the total read counts for each taxon. To use it for species identification, we set a threshold for the total read counts for that species, and then report the species as present if the read count is at least that large. We use the genomes in the TIPP-SD database to build the Metabuli database for the experiments evaluating Metabuli in comparison to TIPP-SD.

#### Metapresence.

Metapresence is used for species detection by mapping metagenomic reads to genomes. To determine whether a species is considered present, it uses a pair of metrics, BER (Breadth-Expected breadth ratio) and FUG (Fraction of Unexpected Gaps) that range from 0 to 1. By specifying values *x* for BER and *y* for FUG, they consider a species to be present only when its *BER* ≥ *x* and *FUG* ≥ *y*. In the Metapresence study [19], the authors did not provide a pre-built database. Hence, for this study, we built customized databases using the procedure described on the Metapresence GitHub page [42]. We tried to create a Bowtie2 database for Metapresence that would include all genomes from the TIPP-SD reference package (∼25k unique species), but the job timed out after 7 days of running time, when given 16 cores and 512 GB of memory, without any execution or out-of-memory errors. Therefore, we built a smaller database using 2000 genomes, with 1000 of the genomes coming from the 1000 known genome dataset and the other 1000 from the TIPP-SD database. The additional 1000 genomes have 80–95% average nucleotide identity (ANI) to at least one of the 1000 known genomes, and ANI was computed using FastANI [43]. Bowtie2 was used to index the genomes when aligning Illumina reads. For aligning PacBio or nanopore reads, we indexed the genomes using Minimap2 with the recommended presets for each type of read.

### Experiments

We performed three experiments.

- Experiment 1: Designing TIPP-SD
- Experiment 2: Comparing TIPP-SD to Bracken, Kraken2, and Metabuli
- Experiment 3: Comparing TIPP-SD to Metapresence

Experiment 1 was performed on the 50 known genomes datasets, and the remaining experiments were performed on the 1000 known genomes datasets and the CAMI II marine datasets.

### Evaluation criteria

We evaluated each method by its ability to detect species that should be present in the input reads, reporting precision and recall. Precision is defined as $\frac{TP}{TP+FP}$, where *TP* is the number of species correctly identified and *FP* is the number of species falsely detected. Recall is defined as $\frac{TP}{TP+FN}$, where *FN* is the number of species that should be detected but are not recovered by the method. We also examined F1 score, which is defined as $\frac{2\cdot precision\cdot recall}{precision+recall}$ and takes both precision and recall into consideration. Precision, recall, and F1 score are all in the range of [0, 1]. We also report AUPR for our precision-recall curves (Table B in S1 Appendix).

To obtain the precision-recall curve of a method, we first extracted the method’s taxonomic identification results. We then used a support threshold to determine if a species was present, and varied the support thresholds to obtain precision-recall curves. The precision-recall curves are computed as follows:

- TIPP-SD: We can use either Marker Vote or Marker Confidence. In each case, we set a threshold *T* between 0 and 1 and say the species is detected if the value is greater than or equal to *T*. By varying *T*, we obtain the precision-recall curve.
- Bracken and Kraken2: These methods can be used for species detection in two ways: the count of reads assigned to a species or the confidence score for the species. As with TIPP-SD, in each case we can set a threshold *X* and report a species as present if the value is greater than or equal to *X*. By varying *X* between 1 and the largest observed value, we obtain precision-recall curves. Bracken and Kraken2 do not have default thresholds for species detection.
- Metabuli: In order to generate our precision and recall curves, we use a threshold *X* for the number of reads assigned at the species level. If a species has at least X reads assigned, then it is considered present in the queryset.
- Metapresence: Recall that Metapresence determines whether a species is considered present based on thresholds *x* for BER (Breadth-Expected breadth ratio) and *y* for FUG (Fraction of Unexpected Gaps) that range from 0 to 1. Thus, given *x* and *y*, Metapresence returns the set of species it detects. To compute precision-recall curves, we compute the corresponding precision and recall value for the pair (*x*, *y*). We compute this for all pairs of (x,y),x∈[0,1],y∈[0,1], with a step of 0.01, and obtain a collection of (precision, recall) pairs. We sort these values by recall in ascending order and then by precision in descending order, and plot them as the Metapresence precision-recall curve.

### Computational limitations

All analyses were performed on the University of Illinois Campus Cluster. Each method was given 900 Gb of memory and 1TB of storage, and limited to 24 hours per input; methods that did not complete within this time were marked as “failures.” Given this limitation, Bracken failed on the 1000 known genomes nanopore datasets due to an intermediary file required for building its database size exceeding 1TB.

## Results

### Experiment 1: Designing TIPP-SD

Recall that TIPP-SD depends on three algorithmic parameters: (1) the technique used to add query sequences into marker gene multiple sequence alignments, (2) the maximum likelihood method used to annotate the edges of a marker gene taxonomic tree for each query sequence mapped to that marker gene, and (3) whether to use marker vote or marker confidence. In this experiment, we examined the impact of each algorithmic parameter using the 50 known genome datasets.

The results of Experiment 1 are provided in detail in Figs A–C in S1 Appendix, and summarized here. First, we found that using marker confidence rather than marker vote improved accuracy. Second, we found that using WITCH to add query sequences into marker gene alignments improved accuracy but increased the runtime substantially, compared to using BLASTN. Third, we found that using pplacer-taxtastic for the maximum likelihood phylogenetic placement method provided the highest accuracy but was also the slowest, BSCAMPP with EPA-ng was the fastest but had the lowest accuracy, while BCAMPP used with pplacer provided nearly the same accuracy as pplacer-taxtastic and was nearly as fast as BSCAMPP with EPA-ng. Based on these results, for the default setting for TIPP-SD we selected BLASTN for adding query sequences into alignments, BSCAMPP with pplacer for the maximum likelihood phylogenetic placement method, and marker confidence. All the remaining experiments use these settings.

#### Selecting default detection threshold.

We examined the F1 score with respect to marker confidence for all datasets and showed the comparisons in Fig D in S1 Appendix. This comparison showed that in general a wide range of threshold values provided good accuracy and that a “conservative” threshold of value ∼0.2 achieves a good F1 score over most datasets for TIPP-SD. For higher recall, TIPP-SD can use a threshold of value ∼0.12. We set these two default values for *T* (one that has higher recall but lower precision, and one that has slightly lower recall and slightly higher precision) and indicate these in the figures as red stars.

### Experiment 2: Comparison to TIPP3, Bracken, Kraken2, and Metabuli

This experiment compared TIPP-SD to TIPP3, Bracken, Kraken2, and Metabuli used for species detection, all of which require the use of a threshold to determine which species are included in the list. Thus, the comparisons were based on precision-recall curves. All methods were compared on the testing datasets.

#### Comparison to TIPP3.

**Fig 1:**
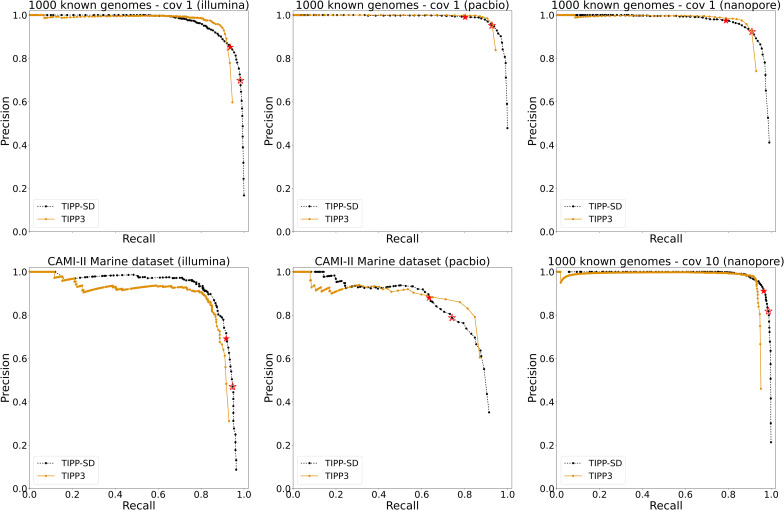
Experiment 2: Precision/recall of TIPP-SD compared to TIPP3. TIPP3’s precision/recall curve is obtained by setting thresholds on the abundance level, and only species with an abundance greater than the threshold are considered as “detected” by TIPP3. Red stars mark the precision and recall values of using the “conservative” (solid) and “sensitive” (hollow) threshold values for TIPP-SD.

We compared TIPP3 to TIPP-SD on all the testing datasets. By varying the threshold for abundance, we obtained precision-recall curves for TIPP3. As seen in Fig 1, TIPP3 had poorer accuracy (in particular, lower recall) than TIPP-SD.

#### How we ran Kraken2, Bracken, and Metabuli.

To use Kraken2, Bracken, and Metabuli for species detection requires that we specify some aspects of the pipeline. For example, we need to determine whether to filter the reads (i.e., remove any reads that do not map to the TIPP-SD marker genes). Each of the methods can be used to state that a species is present based on the number of reads that are assigned to the species (which we refer to as “read voting”), but Kraken2 and Bracken also enable a confidence score to be used. Hence, we must also decide whether to use read voting or confidence scores. We used the 1000 known genomes datasets to determine the best way to run each method.

In evaluating the impact of filtering we determined that filtering was detrimental to all three methods (see Fig E in S1 Appendix). In addition, for Kraken2 and Bracken, we discovered that using confidence improved accuracy compared to read voting (Fig F in S1 Appendix). Therefore, in the remaining experiments, we use each method without filtering, and for Kraken2 and Bracken, we use confidence rather than read voting.

#### Comparison to Kraken2, Bracken, and Metabuli.

**Fig 2:**
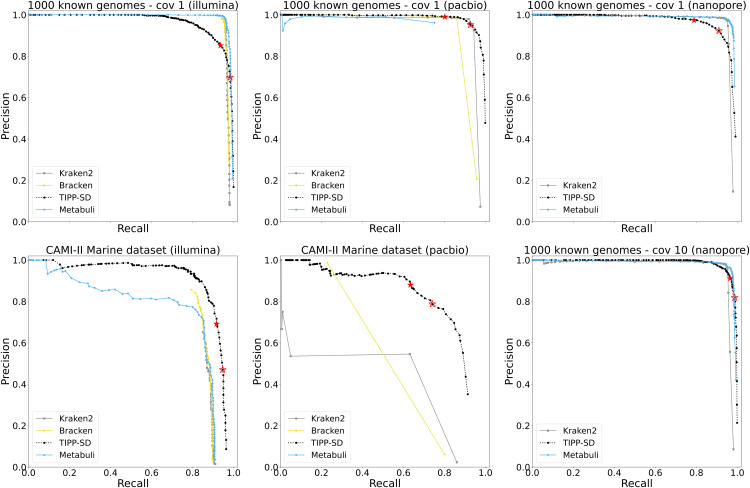
Experiment 2: Comparison of Kraken2, Bracken, Metabuli, and TIPP-SD on all testing datasets. Metabuli did not produce species-level detection for the CAMI II PacBio reads. Bracken was unable to run on the 1000 known genomes nanopore datasets due to computational limitations.

Fig 2 compares TIPP-SD, Kraken2, Bracken, and Metabuli for species detection accuracy using precision-recall curves.

TIPP-SD has better precision and recall than the other methods on the CAMI II datasets and on the 1000 known genomes with PacBio reads. For the two CAMI II datasets, TIPP-SD achieves better precision than all the other methods at all recall levels, and also achieves better recall. On the 1000 known genomes with PacBio reads, TIPP-SD also achieves higher recall than the other methods, but the gap between TIPP-SD and the other methods is not as large as on the CAMI II datasets. However, on the 1000 known genomes with either Illumina or nanopore reads (and for both coverages), one or more of the other methods has better accuracy than TIPP-SD. For example, for 1000 known genomes with Illumina reads, all methods achieve very high recall but differ in precision, with TIPP-SD achieving high recall at lower precision than the other methods.

**Fig 3:**
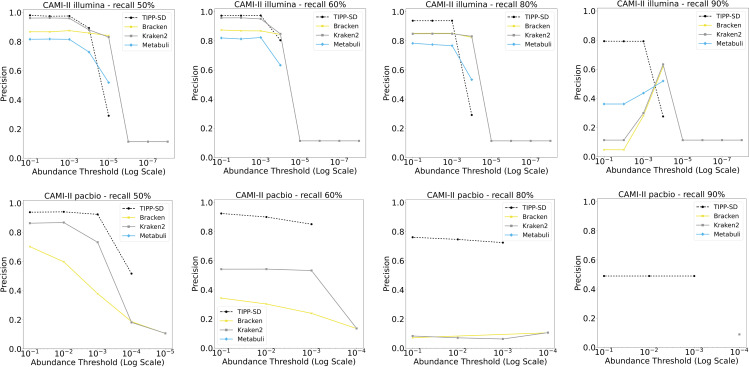
Experiment 2: Precision at different recall levels for low-abundance species detection using Illumina (top) and PacBio (bottom) reads. For each row, from left to right, the subfigures correspond to 50%, 60%, 80%, and 90% recall levels. The x-axis in each subfigure denotes the abundance threshold. Metabuli did not produce species-level detection for the CAMI II PacBio reads.

We then considered precision and recall in recovering low-abundance species, where we specify the maximum allowed abundance for each species. For this question, we examine the CAMI II marine datasets where the estimated species abundance levels vary from very low (0.0000004%) to very high (14.4%). Results on these data are shown in Fig 3. The top row shows results for the Illumina reads and the bottom row shows the results for the PacBio reads, and each row has four subfigures: from left to right, results for the required recall level (50% up to 90%). For each specified recall level and abundance threshold level, we calculated the precision of the set of species detected by the method.

These results show that TIPP-SD achieves higher recall (90%) than all the other methods on the PacBio reads, and when another method does succeed at recovering low abundance species at a lower recall level, TIPP-SD has much better precision. This indicates a strong advantage for TIPP-SD over the other methods for PacBio reads in terms of recovering low abundance species. TIPP-SD also has some advantages over the other methods on the Illumina reads: it achieves substantially better precision for the high recall settings, provided that the tested abundance level is not too low. For the lowest abundance levels, Kraken2 is better than TIPP-SD in that it can achieve higher recall, but it does this with very low precision.

#### Analysis of false positives.

We used two recall thresholds (90% and 95%) to extract the list of reported species by Kraken2, Bracken, Metabuli, and TIPP-SD. We then identified the species falsely reported as present. For each false positive species, we found the closest species (using ANI) in the input to the false positive species, and reported that ANI for the false positive species using FastANI.

**Fig 4:**
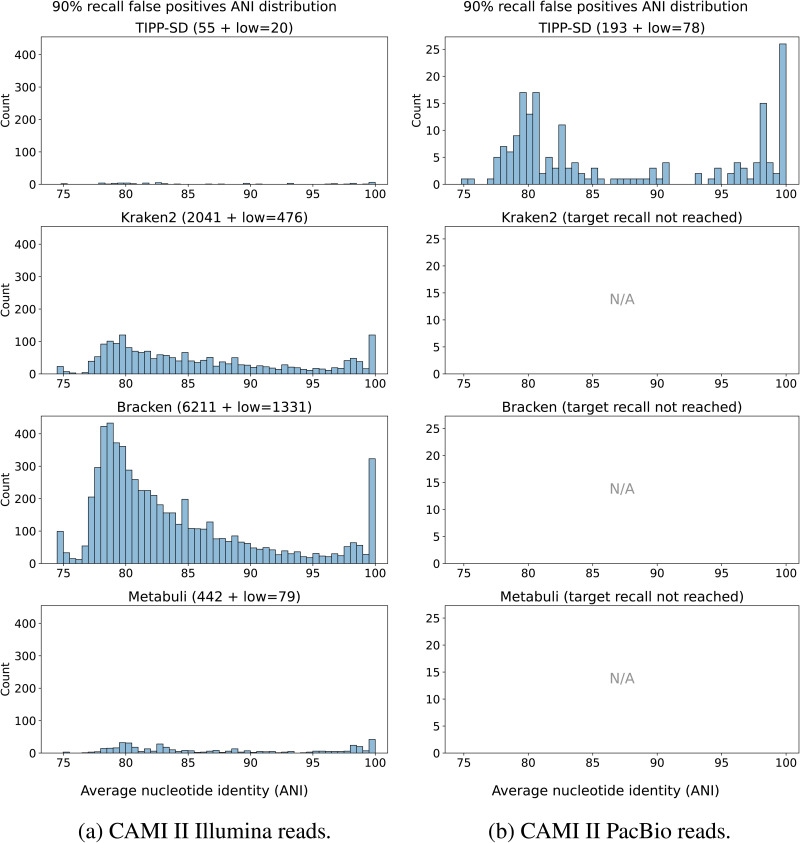
Experiment 2: Analysis of false positives at 90% recall on the CAMI II marine datasets. The distribution of average nucleotide identity (ANI) of false positive species to their closest species in the set of species from the CAMI II dataset (Illumina reads on the left and PacBio reads on the right), for Kraken2, Bracken, Metabuli, and TIPP-SD. The total number of false positives for each method is shown in parentheses, with *low* = *X* meaning that there are *X* false positives that do not have any close species in the target (i.e., ≪80% ANI according to FastANI). Methods that failed to reach the target 90% recall are marked as “N/A”.

On the CAMI II marine datasets, only TIPP-SD achieves 95% recall for the Illumina reads, but no method achieves 95% recall on the PacBio reads (Fig G in S1 Appendix). Therefore, we instead discuss false positives given 90% recall. Only TIPP-SD achieves 90% recall for the PacBio reads (Fig 4). All methods achieve 90% recall on the CAMI II marine dataset using Illumina reads but differ however in the number of false positives: TIPP-SD has the fewest false positives (only 55), followed by Metabuli (442), Kraken2 (2041), and Bracken (6211). Thus, on the CAMI II marine datasets, TIPP-SD has superior accuracy in terms of false positives when it achieves high recall.

**Fig 5:**
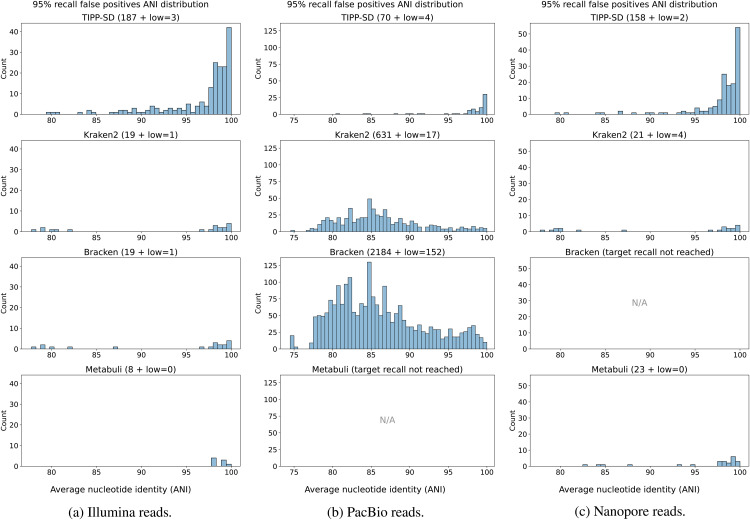
Analysis of false positives given 95% recall on 1000 known genomes, coverage 1. At 95% recall, the distribution of average nucleotide identity (ANI) of false-positive species to their closest species in the set of 1000 known genomes, for Kraken2, Bracken, and TIPP-SD classifying Illumina (left), PacBio (middle), and nanopore (right) reads. The total number of false positives for each method is shown in parentheses, with *low* = *X* meaning that there are *X* false positives that do not have any close species in the target (i.e., ≪80% ANI according to FastANI). Methods that failed to reach the target 95% recall are marked as “N/A”.

Results for 95% recall on the 1000 known genomes with coverage 1 are shown in Fig 5. On the PacBio reads, all methods other than Metabuli achieved 95% recall; on these data, TIPP-SD had the smallest number of false positives (70), followed by Kraken2 (631), and then Bracken (2184). On the Illumina reads, all methods achieved 95% recall, but differed in the number of false positives; Metabuli had only 8, Kraken2 and Bracken both had 19, and TIPP-SD had 187. On the nanopore reads, all methods except Bracken achieved 95% recall and again differed in the number of false positives: Kraken2 had the fewest (21), Metabuli had 23, and TIPP-SD had 158. Results for 90% recall show similar relative trends but with lower numbers of false positives, and are shown in Fig H in S1 Appendix.

#### Computational performance.

Fig I in S1 Appendix compares the runtime of TIPP-SD, Bracken, Kraken2, and Metabuli. Recall that each method had access to 64 cores, except for TIPP-SD which used only 16 cores. For the 1000 known genomes nanopore datasets, Bracken failed to complete because its database exceeded our memory limitations (1TB), otherwise all methods completed on all datasets within our computational limits. On all the datasets, the relative runtimes were as follows: TIPP-SD was the slowest, followed by Metabuli, and then by Kraken2 and Bracken (which were close in runtime). Nevertheless, TIPP-SD was still fast enough to complete on all datasets in 0.5–8.6 hours with 16 CPU cores. The runtime breakdown on alignment and placement for TIPP-SD can be found in Table C in S1 Appendix. Each step contributes relatively equally to the runtime, although the BLASTN step takes more time than the placement step on the Illumina read datasets, the the placement step takes more time than the BLASTN step on the long read datasets. The BLASTN step takes 0.5–4.2 hours, positively correlated with the number of reads in the input, and the placement time takes 0.8–4.3 hours, affected by the number of reads being placed, the alignment length, and the number of genomes in the reference taxonomies.

The methods differed in terms of memory usage (Fig J in S1 Appendix). Kraken2 and Bracken had the highest memory usage, exceeding 300Gb on the long read datasets and even using ∼600Gb on the nanopore 1000 genome dataset with coverage 10. Metabuli had the next highest memory usage, ∼100Gb or more for all datasets. TIPP-SD had much lower memory usage, using 3–29 GBs for all datasets.

### Experiment 3: Comparison to Metapresence

**Fig 6:**
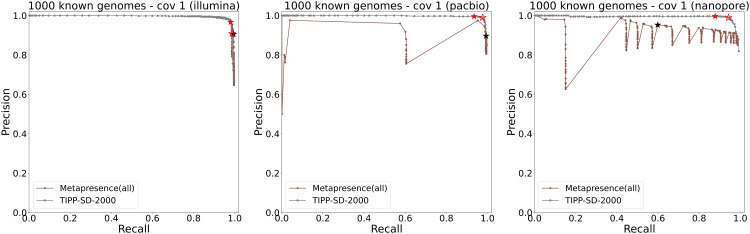
Experiment 3: Precision-recall curves of Metapresence and TIPP-SD-2000. We use the TIPP3 reference package restricted to the same 2000 species as in Metapresence on 1000 known genome datasets with Illumina, PacBio, and nanopore reads. The black star in each subfigure shows the precision and recall using the Metapresence default threshold (i.e., a species is considered present if and only if *BER* ≥ 0.8 and *FUG* ≥ 0.5), while the remaining data points are results from a grid search of different *BER* and *FUG* values. The red solid and hollow stars show the precision and recall of TIPP-SD-2000 using the “conservative” and “sensitive” thresholds, respectively.

For this experiment, we restricted the reference package for TIPP-SD to the same set of 2000 species, and refer to this modification as TIPP-SD-2000. Fig 6 shows the precision-recall curves of TIPP-SD-2000 and Metapresence on the three 1000 known genomes datasets. For Illumina reads, TIPP-SD-2000 and Metapresence were similar in accuracy, and both achieved very high precision and recall. For the long read datasets, Metapresence achieved the same very high recall as TIPP-SD-2000 but with lower precision.

Precision and recall of Metapresence using its default threshold values (i.e., *BER* = 0.8,*FUG* = 0.5) are shown in each of the subfigures as black stars. Red solid and hollow stars show the precision and recall of TIPP-SD-2000 using the “conservative” and “sensitive” thresholds, respectively. Using these default thresholds for the two methods, Metapresence was identical for precision and recall to TIPP-SD-2000 given its sensitive threshold on the Illumina reads, slightly better for recall but lower for precision on the PacBio reads for both TIPP-SD-2000 thresholds, and lower in both precision and recall on the nanopore reads for both TIPP-SD-2000 thresholds.

We also compared the runtime and memory usage between TIPP-SD-2000 and Metapresence, on the three datasets of 1000 known genomes with Illumina, PacBio, or nanopore reads (Fig K in S1 Appendix). Metapresence is substantially faster than TIPP-SD-2000 for all three datasets but uses more memory than TIPP-SD-2000 on the long read datasets.

## Discussion and conclusions

These results show that TIPP-SD has a reliable advantage over Kraken2, Bracken, and Metabuli on the CAMI II datasets and on PacBio reads from the 1000 known genome dataset. Interestingly, TIPP-SD is not as accurate as the best alternative method on the 1000 known genome datasets with Illumina or nanopore reads. The comparison to Metapresence was only performed on a small subset of the reference for the 1000 known genomes datasets, and for these data, TIPP-SD was tied on Illumina and had an advantage on the PacBio and nanopore reads.

Hence, these results reveal that absolute and relative accuracy of methods differs between the CAMI II marine datasets and the 1000 known genome datasets. While the driving factor for this difference requires further investigation, a likely explanation is that the CAMI II datasets have species abundances that vary widely, while every species has the same frequency in the 1000 known genomes datasets. This difference may suggest that results on the CAMI II datasets are more representative of performance of real-world datasets than the 1000 known genome datasets.

It is worth noting the high accuracy for both Metapresence and TIPP-SD-2000 reported in Experiment 3, which was based on the 1000 known genome datasets. We conjecture that this may be due to the small number of genomes (only 2000) used in this experiment, since [44] noted that database size positively correlates with the loss of resolution at the species level. Here we note that our attempt to build a large database for Metapresence containing all of the genomes used in the TIPP-SD database ran out of memory and timed out after 7 days; hence, assessing Metapresence’s accuracy given larger databases is important but logistically challenging.

Overall, we find that TIPP-SD has an advantage over the other methods we examined under conditions with highly variable abundance levels (as in the CAMI II datasets) or sequencing error (as in the PacBio reads). This advantage may be due to its ability to use maximum likelihood phylogenetic placement and highly accurate sequence alignments, rather than relying on k-mers (in the case of Kraken2, Bracken, and Metapresence) and meta-mers (Metabuli).

We find that TIPP-SD is slower than the competing methods, due to its algorithmic approach (both BLASTN and BCAMPP(p) can be expensive); nevertheless, it is fast enough to run on large input data (∼36 GBs in FASTA format) in under 10 hours. Furthermore, TIPP-SD has low memory requirements, much lower than the other methods, whose memory requirements can be very high (nearing 1TB). Overall, TIPP-SD is a competitive method with both reasonable speed and high accuracy for species detection.

This study suggests several directions for improving TIPP-SD. Most importantly, we found that Kraken2, Bracken, and Metabuli benefitted by not filtering reads. Hence, to improve accuracy, we should examine the impact of not filtering as many reads, which might require modifications to TIPP-SD algorithmically. Since TIPP-SD is designed to add reads into marker gene alignments and then place them into taxonomic trees based on the marker genes, TIPP-SD can easily be extended to work with reads that map to genes that are not marker genes (i.e., not single copy or universal). To work with reads coming from genes that are not marker genes, we would need to build multiple sequence alignments and taxonomic trees for each added gene; this would require some additional development and expansion of the reference package but is completely feasible. The runtime and memory usage would increase but nevertheless might improve accuracy. Improvements to the implementation, and the development of a better parallel implementation (perhaps a distributed computing implementation) would improve the running time and could make TIPP-SD competitive for running time with the other methods.

Another design change that could lead to an improvement in accuracy is to reconsider how we set the default marker confidence thresholds: in this study we picked these defaults for TIPP-SD without considering the sequencing technology, and yet our analyses show that thresholds that are best for may differ between Illumina, PacBio, and nanopore; hence, a more careful setting of the default thresholds that take dataset properties into account is advisable.

We showed results for TIPP-SD for species detection, but future work should examine results for detecting other taxonomic levels, such as genera, families, or even strains. Although TIPP-SD (and the other members of the TIPP family of methods) have been developed for microbiome analysis, we could extend TIPP-SD to analyze viral sequences, which would enable its use in other applications. Finally, TIPP-SD should be compared to other methods that have been developed for species detection.

Other future work includes exploring its use in newer sequencing technologies. Our evaluation of PacBio reads with high error rates is helpful in understanding robustness to high sequencing error, but the new PacBio reads have much lower sequencing error rates [45,46]. Similarly, the newer nanopore technology also has somewhat lower sequencing error than what we examined in our study [47]. We also note that hi-fi reads are increasingly of interest [48]. Thus, future work should examine TIPP-SD under these new sequencing technologies.

The experiments we performed revealed the importance of testing under conditions that are heterogeneous in terms of abundance levels, and so additional experiments should be performed on datasets with varying abundance levels and taxonomic sampling strategies.

## Supporting information

S1 Appendix All supporting text, 11 supplementary figures, and 4 supplementary tables are included. (PDF)
