# Supplementary material for: TIPP-SD: A new method for species detection in microbiomes
Source: PLoS Comput Biol. 2026 May 28;22(5):e1014347. doi: 10.1371/journal.pcbi.1014347 (PMC13229341; doi:10.1371/journal.pcbi.1014347)
Supplement: S1 Appendix — (PDF) [file pcbi.1014347.s001.pdf]

Appendix for "TIPP-SD: a new method for species detection in microbiomes"

Chengze Shen, Eleanor Wedell, Mihai Pop, and Tandy Warnow

Contents

A Additional details on experiment design **3**
A.1 TIPP3 reference package raw data 3
A.2 Software commands 3
A.3 Additional comments 5

B Additional results **6**
B.1 Results for Experiment 1 6
B.2 Additional results for Experiment 2 9
B.2.1 Filtering vs. all reads for Kraken2, Bracken, and Metabuli 9
B.2.2 Voting vs confidence for Kraken2 and Bracken 10
B.2.3 AUPR 11
B.2.4 Analysis of low-abundance species detection 12
B.2.5 Analyses of false positives 13
B.2.6 Runtime and memory 15
B.3 Additional results for Experiment 3 17

List of Figures

A Experiment 1: Precision-recall for marker vote vs marker confidence in one variant of TIPP-SD . . . 6
B Experiment 1: Precision/recall curves of TIPP-SD variants using marker confidence 7
C Experiment 1: Runtime and memory of TIPP-SD variants using marker confidence . 8
D Experiment 1: Relationship between marker confidence threshold and F1 score . . . 8
E Experiment 2: Evaluating impact of filtering on accuracy of Kraken2, Bracken, and Metabuli . . . 9
F Experiment 2: Voting vs Confidence for Kraken2 and Bracken . . . 10
G Experiment 2: Analysis of false positives on CAMI-II genomes, 95% recall . . . 13
H Experiment 2: Analysis of false positives at 90% recall for the 1000 known genome datasets . . . 14
I Experiment 2: Runtime of TIPP-SD, Kraken2, Bracken, and Metabuli . . . 15
J Experiment 2: Memory usage of TIPP-SD, Kraken2, Bracken, and Metabuli . . . 16
K Experiment 3: Runtime and memory for TIPP-SD-2000 and Metapresence . . . 17

List of Tables

A Parameter settings for each TIPP-SD variant. . . . 4
B Experiment 2: AUPR for Method Comparison . . . 11
C Experiment 2: Runtime breakdown on alignment and placement in hours for TIPP-SD. 16
D Experiment 3: AUPR for TIPP-SD-2000 vs. Metapresence . . . 17

A Additional details on experiment design

A.1 TIPP3 reference package raw data

TIPP-SD used the TIPP3 reference package, which was generated from the lists of assemblies (Bacteria and Archaea) downloaded in TIPP2, and marker gene sequences were collected using FetchMG.

We ran Kraken2 (v2.17.1) and Bracken (v2.9) with custom databases built using the same genomes contained in the TIPP3 reference package. For Illumina style reads we used the default settings, but for Pacific Biosciences style reads and nanopore ONT reads we used a k-mer length of 26 (as recommended by [5]) and the following commands to build the Kraken and Bracken databases:

```
$ kraken2-build --build \
                --db [Nanopore and PacBio database] \
                --threads 64 \
                --kmer-len 26 \
                --minimizer-len 26 \
                --minimizer-spaces 6

$ kraken2-build --build \
                --db [Illumina database] \
                --threads 64

$ bracken-build -d [Bracken illumina database] -t 64 -k 35 -l 150

$ bracken-build -d [Bracken PacBio database] -t 64 -k 26 -l 400
```

When attempting to build a database for Bracken with the appropriate read length for nanopore style reads, an intermediary file (Kraken.report that compares all reads to all genomes in the dataset) was created whose size exceeded 1TB before completion. Thus, we have excluded Bracken for our analysis of nanopore style reads.

We also use a database that we built in our study of TIPP3 [6] for Metabuli, which also uses identical genomes to those used by TIPP-SD.

A.2 Software commands

All software are given a maximum of 64 cores and 900 GB of memory to run until completion.

1. We ran Kraken2 (v2.17.1) with the following command in order to generate results with a given confidence value given as an input parameter:

```
$ kraken2 --db [kraken2 database] \
           [query reads file] \
          --threads 64 \
          --output [kraken2.output] \
          --report [kraken2.report] \
          --confidence [confidence -- we varied from 0, to .98 by .02]
```

2. To use Bracken (v3.0.1) with a given confidence value, we first ran Kraken2 with that value (using the command in the previous paragraph), to produce the Kraken2 report. We then gave this report to Bracken. In this way, we use the confidence value to generate the input to Bracken. The command we used is as follows:

```
$ bracken -r [read length (150 for Illumina, 400 for Pacific Biosciences)] \
          -d [Bracken database] \
          -i [Kraken2 report] \
          -w [Bracken report] \
          -o [Bracken output]
```

3. We ran Metabuli (v1.1.1) using the following commands

```
$ metabuli classify \
         --seq-mode [1 for Illumina, 3 for PacBio and nanopore] \
         --min-score [0.15 for Illumina, 0.07 for PacBio and nanopore] \
         --min-sp-score [0.5 for Illumina, 0.3 for PacBio and nanopore] \
         [query reads file] \
         [Metabuli database] \
         [output directory] \
         metabuli_job \
         --threads 64
```

4. We ran TIPP-SD (v0.3) and its variants with the following commands. `--alignment-method` controls what method we used to align the reads, and `--placement-method` and `--bscampp-mode` control what method we used for read placement.

```
$ run_tipp3.py abundance -i [query reads file] \
    -r [reference package directory] --outdir [output directory] \
    --alignment-method XXX \
    --placement-method YYY \
    --bscampp-mode ZZZ \
    -t 16
```

Table A: Parameter settings for each TIPP-SD variant.

Method	`--alignment-method`	`--placement-method`	`--bscampp-mode`
Variant A (TIPP-SD)	BLAST	BSCAMPP	pplacer
Variant B	WITCH	pplacer-taxtastic	-
Variant C	BLAST	BSCAMPP	EPA-ng

5. For Metapresence (v1.0), we first aligned the input reads, where the alignment tool depended on the sequencing technology. We used Bowtie2 (v2.5.4) [3] and samtools (v1.19.2) [1] when dealing with Illumina reads, and Minimap2 (v2.28-r1209) [4] when dealing with PacBio and nanopore reads.

```
$ bowtie2 -f [query reads file] -x [bowtie2 database index] -p 16 \
    | samtools view -b -@ 16 \
    | samtools sort -@ 16 > [bam alignment file]
```

We then indexed the bowtie2 alignment with samtools:

```
$ samtools index -@ 16 [bam alignment file]
```

In the case of PacBio and nanopore reads, we used minimap2 for indexing and alignment (`-H` for PacBio index). `map-pb` was used for PacBio, and `map-ont` was used for nanopore.

```
#### for indexing
$ minimap2 [-H] -x [map-pb|map-ont] -t 16 -I 256G \
    -d minimap_ref_[pacbio|nanopore].mmi [all genome file]

```

```
#### for alignment
$ minimap2 -ax [map-pb|map-ont] -t 16 \
    minimap_ref_[pacbio|nanopore].mmi [query reads file] \
    | samtools view -b -@ 16 \
    | samtools sort -@ 16 > [bam alignment file]

#### for samtools index
$ samtools index -@ 16 [bam alignment file]
```

Finally, we ran Metapresence to analyze the alignment results:

```
$ metapresence.py [reference genomes directory] [bam alignment file] \
    -o [output prefix] -p 16
```

6. To compute average nucleotide identity (ANI) between two sets of genomes, we used fastANI (v1.34) [2] and the following command. Note that fastANI does not return comparisons that have $\ll 80\%$ ANI.

```
$ fastANI --ql [query list of genomes] --rl [target list of genomes] \
    -o [output file] -t 16
```

A.3 Additional comments

Kraken2 and Bracken were run and evaluated in two different ways to generate precision/recall curves, one based on the number of reads assigned to a species and the other based on the confidence score, as we now describe. See items (1) and (2) in Sect A.2, above, to understand the protocol by which we use a confidence threshold.

For the first way, which we call 'vote_Kraken2' and 'vote_Bracken', we use a confidence score of 0.0 with the previous commands and vary a threshold X based on the number of reads assigned to a species. If the number of reads is at least X, then a species is considered present. The Bracken report generated using the 0.0 confidence level Kraken2 report is processed in the same way. Now each point along our precision/recall curve corresponds to a particular threshold level.

For the second way, which we refer to as 'conf_Kraken2' and 'conf_Bracken', we vary the confidence score from 0 to 1 in increments of 0.02 and consider every reported species at a given confidence level as present. We also generate a new Bracken report from each Kraken2 report generated for a given confidence level, and all species listed in a given Bracken report are considered present. For this way of running Kraken2 and Bracken, each point along our precision/recall curve corresponds to a different run of Kraken2 and Bracken.

B Additional results

B.1 Results for Experiment 1

TIPP-SD has several variants that differ by how the query sequences are added into the marker gene alignments, which phylogenetic placement method is used, and whether we use Marker Vote or Marker Confidence. In selecting the default setting for TIPP-SD, we were informed by trends observed in two previous studies: the study designing TIPP3 [6] and the study designing BSCAMPP [7]. The TIPP3 study presented a fast version of TIPP3, called TIPP3-fast, that used BLASTN for aligning reads and EPA-ng with BSCAMPP, for phylogenetic placement. This variant had close to the accuracy of TIPP3, which used a more accurate alignment method (WITCH) and a more accurate phylogenetic placement method (pplacer-taxtastic). However, the later study [7] showed that using BSCAMPP with pplacer was more accurate than BSCAMPP with EPA-ng and was only slightly slower. Hence, in this study, we use, as a starting point in our search for a good default setting, the variant where we use BLASTN for the alignment step and BSCAMPP with pplacer for the phylogenetic placement method. We call this Variant A. We then performed a sequence of experiments.

In our first analysis, we evaluated the choice of Marker Vote or Marker Confidence using Variant A, which we note relies on BLASTN and BSCAMPP(p). As seen in Fig A, using marker confidence improved accuracy compared to using marker vote.

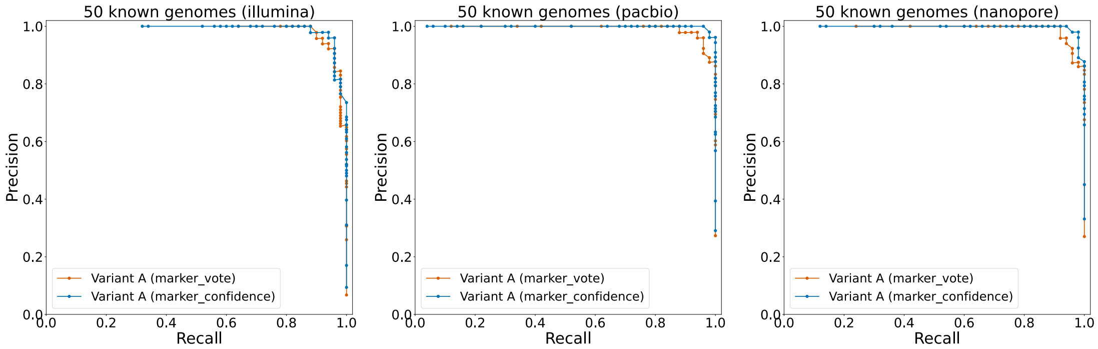

Fig A: **Experiment 1: Precision-recall for marker vote vs marker confidence in one variant of TIPP-SD.** The two variants of TIPP-SD both use BLASTN for read alignment and BSCAMPP(p) for phylogenetic placement, but differ in the final step: using either marker vote or marker confidence. This experiment was performed on the algorithm design benchmark datasets.

In our second analysis, we examined two other variants of TIPP-SD that use different read alignment and phylogenetic placement methods, while keeping marker confidence. Variant B uses WITCH for aligning reads and pplacer-taxtastic for placement (i.e., the same setting as for TIPP3), and Variant C uses BLASTN for aligning reads and BSCAMPP with EPA-ng for placement (i.e., the same setting as TIPP3-fast).

As seen in Fig B, Variant C was the least accurate method, and Variant B was the most accurate. On the other hand, Variant A was much faster and had lower memory usage than Variant B (Fig C), with the runtime improvement typically by 1-2 orders of magnitude. Given that the accuracy improvement obtained by Variant B over Variant A was small and the increase in runtime and memory usage was very large, we selected Variant A as the default for TIPP-SD for species detection. Thus, the default setting is:

- BLASTN for read alignment
- BSCAMPP with pplacer for phylogenetic placement
- Marker Confidence for the final step

We refer to this default as "TIPP-SD", and we report results using these settings in subsequent experiments.

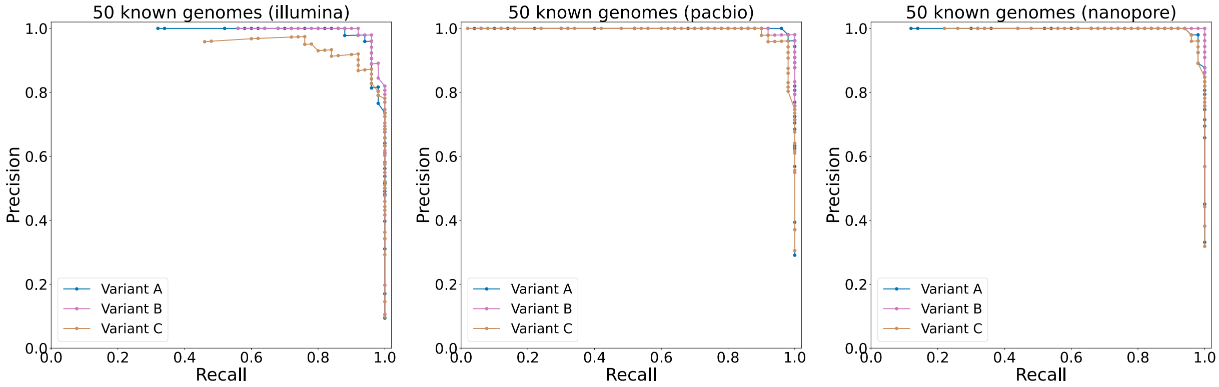

Fig B: **Experiment 1: Precision/recall of TIPP-SD Variants A, B, and C, each using marker confidence for the final step.** Variant A uses BLASTN for read alignment and BSCAMPP(p) for phylogenetic placement of reads into the taxonomy for the marker gene; Variant B uses WITCH for aligning reads and pplacer-taxtastic for placement; and Variant C uses BLASTN for aligning reads and BSCAMPP with EPA-ng for placement.

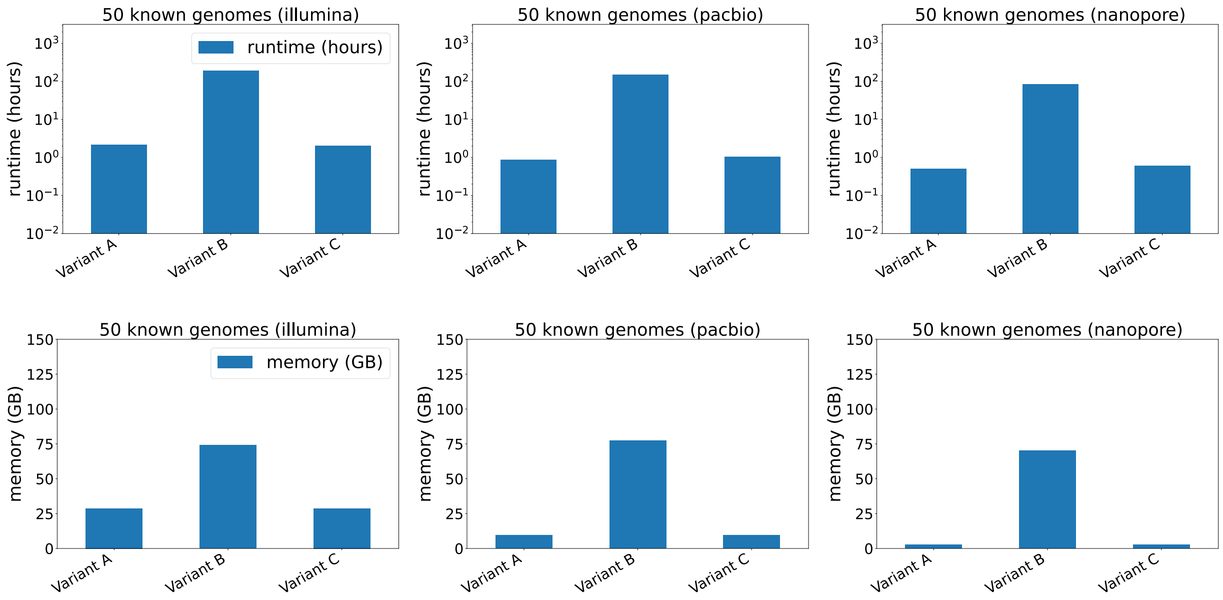

Fig C: **Experiment 1: Runtime (top) and memory (bottom) in hours (in log-scale) of three variants of TIPP-SD, each using marker confidence.** Variant A uses BLASTN for read alignment and BSCAMPP(p) for phylogenetic placement of reads into the taxonomy for the marker gene; Variant B uses WITCH for aligning reads and pplacer-taxtastic for placement; and Variant C uses BLASTN for aligning reads and BSCAMPP with EPA-ng for placement.

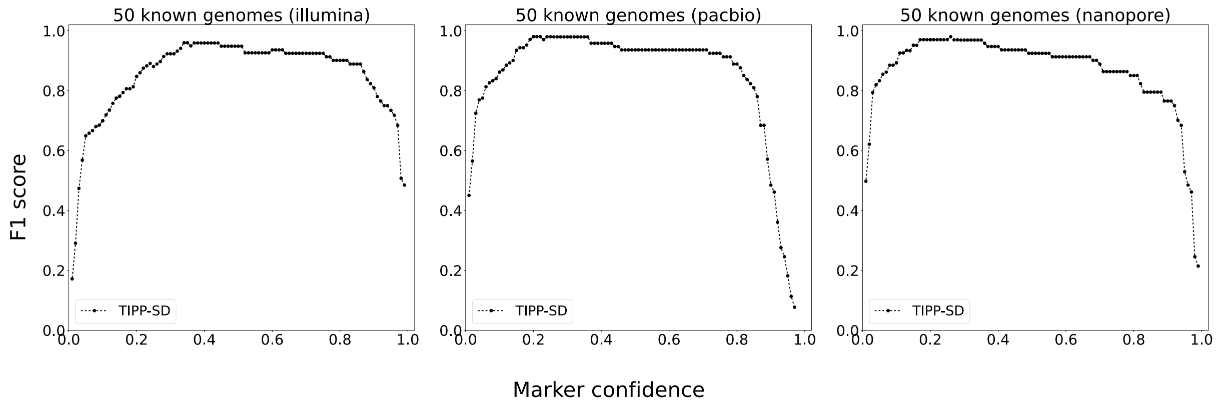

Fig D: **Experiment 1: Relationship between marker confidence and F1 score of TIPP-SD on the algorithm design datasets.**

We then set out to select default values for the threshold used by TIPP-SD. Fig D shows the relationship between marker confidence threshold and F1 score of TIPP-SD on the 50 known genome datasets. In general, a wide range of marker confidence values leads to high F1 scores, suggesting that the marker confidence strategy is fairly robust. However, the peak F1 score happened when marker confidence was 0.3–0.6 for Illumina reads, 0.2–0.3 for PacBio reads, and 0.2–0.3 for nanopore reads.

The overall trends suggest that a threshold around 0.2 should be a good setting for most conditions. However, for Illumina reads, larger thresholds (e.g., around 0.6) provide better results.

B.2 Additional results for Experiment 2

B.2.1 Filtering vs. all reads for Kraken2, Bracken, and Metabuli

Fig E evaluates the impact of filtering on Kraken2, Bracken, and Metabuli using the 1000 known genomes with coverage 1 datasets. For all three methods, filtering hurt accuracy (and so using all reads improved accuracy).

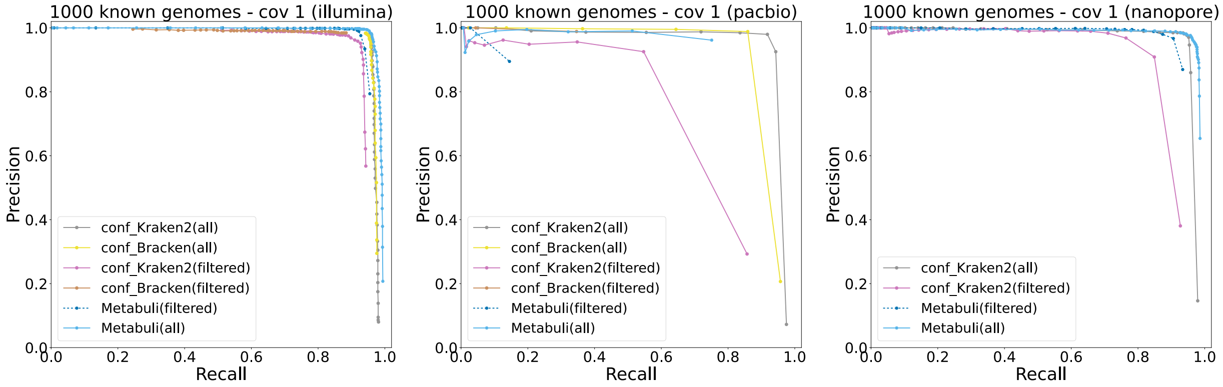

Fig E: **Experiment 2: Impact of filtering on accuracy for Kraken2, Bracken, and Metabuli.** Analyses without filtering are indicated by "(all)". Bracken was not run for nanopore reads due to computational limitations for building the database with long reads. Bracken(filtered) for PacBio reads was run but achieves recall at most 0.4 (which matches Kraken(all) at this point), and hence almost cannot be seen.

B.2.2 Voting vs confidence for Kraken2 and Bracken

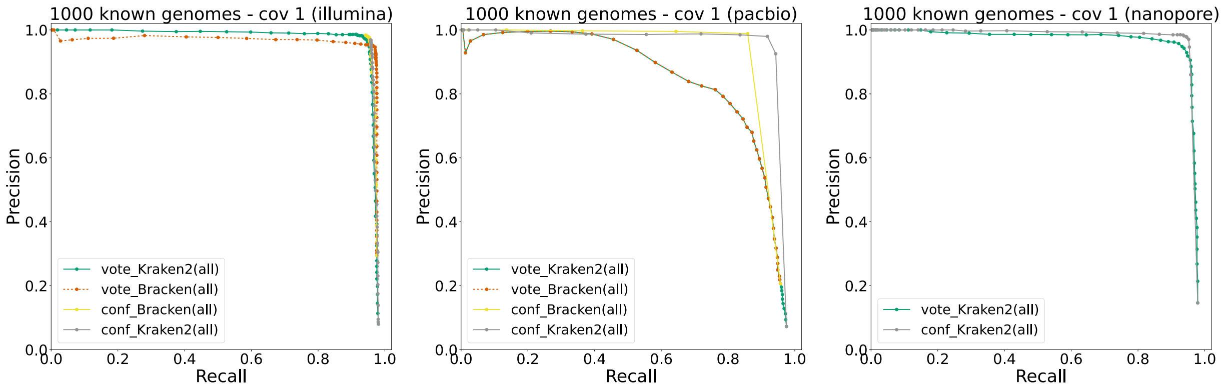

Fig F: **Experiment 2: Voting vs. confidence for Kraken2 and Bracken.** Bracken was not run for nanopore reads due to computational limitations for building the database with long reads.

We used the 1000 known genomes with coverage 1 datasets to compare voting and confidence techniques for species detection for Kraken2 and Bracken. As seen in Fig F, for both Kraken2 and Bracken, while both voting and confidence can result in high recall, only using confidence rather than voting also yields high precision.

B.2.3 AUPR

We report AUPR values in Table B. These AUPR values should be interpreted with care, as they can be somewhat misleading for this context. That is, species detection methods are used generally only for settings that have high recall (90% or higher if possible), and then within that high recall setting they seek to have good precision. AUPR instead addresses the entire range. This can penalize methods that only return high recall values compared to methods that can return lower as well as high recall values (e.g., Bracken and Kraken2 have low AUPR values for CAMI II Illumina and yet have very good accuracy in that setting). It also does not recognize when a method is able to identify default values that provide high recall (e.g., TIPP-SD for the 1000 Genomes with nanopore reads, coverage 10).

Table B: **Experiment 2: AUPR for precision-recall curves of each method on all testing querysets.** These AUPR values are for the results shown in Fig 2 in the paper. An 'X' indicates that the method failed to return any results. A '-' indicates that the method was unable to run within our computational limitations.

Queryset	1000 Genomes Illumina (Cov1)	1000 Genomes PacBio (Cov1)	1000 Genomes Nanopore (Cov1)	CAMI II Illumina	CAMI II PacBio	1000 Genomes Nanopore (Cov10)
TIPP-SD	0.965	0.985	0.963	0.832	0.782	0.931
Kraken2	0.015	0.946	0.962	0.023	0.410	0.959
Bracken	0.029	0.912	-	0.070	0.299	-
Metabuli	0.984	0.732	0.977	0.757	X	0.980

B.2.4 Analysis of low-abundance species detection

We also examined how TIPP-SD, Kraken2, Bracken, and Metabuli perform on the detection of low-abundance species. We define low-abundance species as the species from the target sample with $\leq X$ abundance (i.e., **abundance threshold**), in which we can vary X to obtain each method's precision and recall at different levels of "low abundance".

For each low-abundance level X, we parse each method's detected species and sort them by the following rules:

1. TIPP-SD: Detected species are sorted by "marker_confidence" from high to low for species detection.

2. Kraken2 and Bracken (confidence): As noted in (1) and (2) in Sect A.2, Kraken2 allows a parameter setting of "confidence" when operating on input reads, and only the detected species passing the confidence threshold are reported. To provide a confidence value to Bracken, we use that value as input to Kraken2, and then pass the Kraken2 report as input to Bracken. To generate the precision/recall curves based on confidence, we varied the confidence threshold between 0 and 1, incrementing by 0.02. We then sort the species by their largest appearing confidence threshold. For example, if a species was reported by Bracken with confidence threshold 0.2, but was not reported at confidence threshold 0.22, then the largest appearing confidence threshold for this species is 0.2.

3. Metabuli: Each species is annotated by the number of reads that are assigned to the species. We sort the species by the number of reads assigned to the species.

We use the sorted list of species of each method to obtain its recall of low-abundance species. For a method, we include all species from the sorted species list until we achieve the target recall level of the set of low-abundance species for each target level X. We then compute the overall precision of the corresponding method. If a method fails to achieve the target recall level for the low-abundance species, its data point will not be present in the figure.

B.2.5 Analyses of false positives

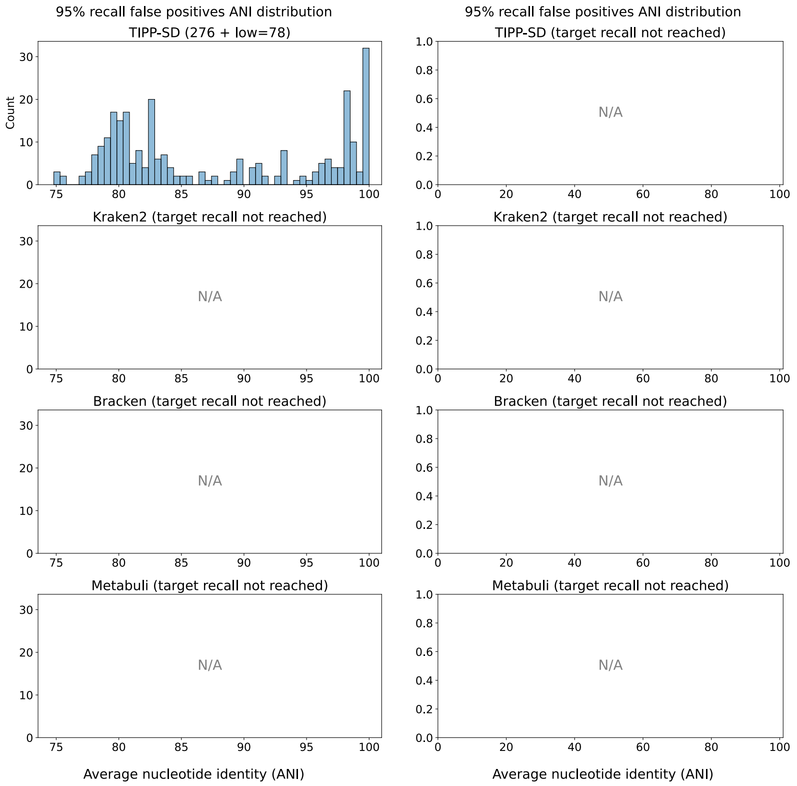

(a) CAMI-II Illumina reads (b) CAMI-II PacBio reads

Fig G: **Experiment 2: Analysis of false positives at 95% recall on the CAMI-II genome datasets.** The distribution of average nucleotide identity (ANI) of false positive species to their closest species in the set of species from the CAMI-II dataset (Illumina reads on the left and PacBio reads on the right), for Kraken2, Bracken, Metabuli, and TIPP-SD. The total number of false positives for each method is shown in parentheses, with $low = X$ meaning that there are X false positives that do not have any close species in the target (i.e., $\ll 80\%$ ANI according to fastANI). Methods that failed to reach the target 95% recall are marked as "N/A".

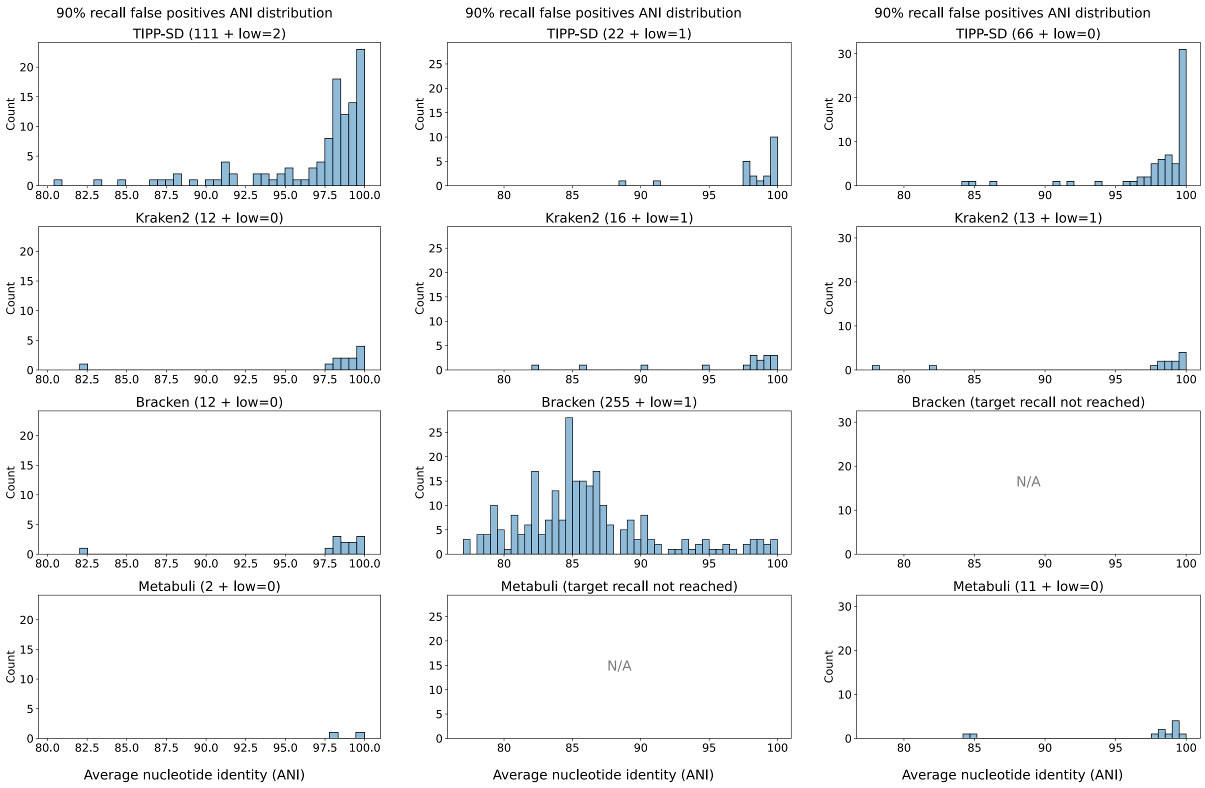

(a) Illumina reads.

(b) PacBio reads.

(c) Nanopore reads.

Fig H: **Experiment 2: Analysis of false positives at 90% recall on the 1000 known genome datasets.** The distribution of average nucleotide identity (ANI) of false positive species to their closest species in the set of species from the 1000 known genome dataset. Results are shown for Kraken2, Bracken, and TIPP-SD classifying Illumina (left), PacBio (middle), and nanopore (right) reads. The total number of false positives for each method is shown in parentheses, with $low = X$ meaning that there are X false positives that do not have any close species in the target (i.e., $\ll$ 80% ANI according to fastANI). Methods that failed to reach the target 90% recall are marked as “N/A”.

B.2.6 Runtime and memory

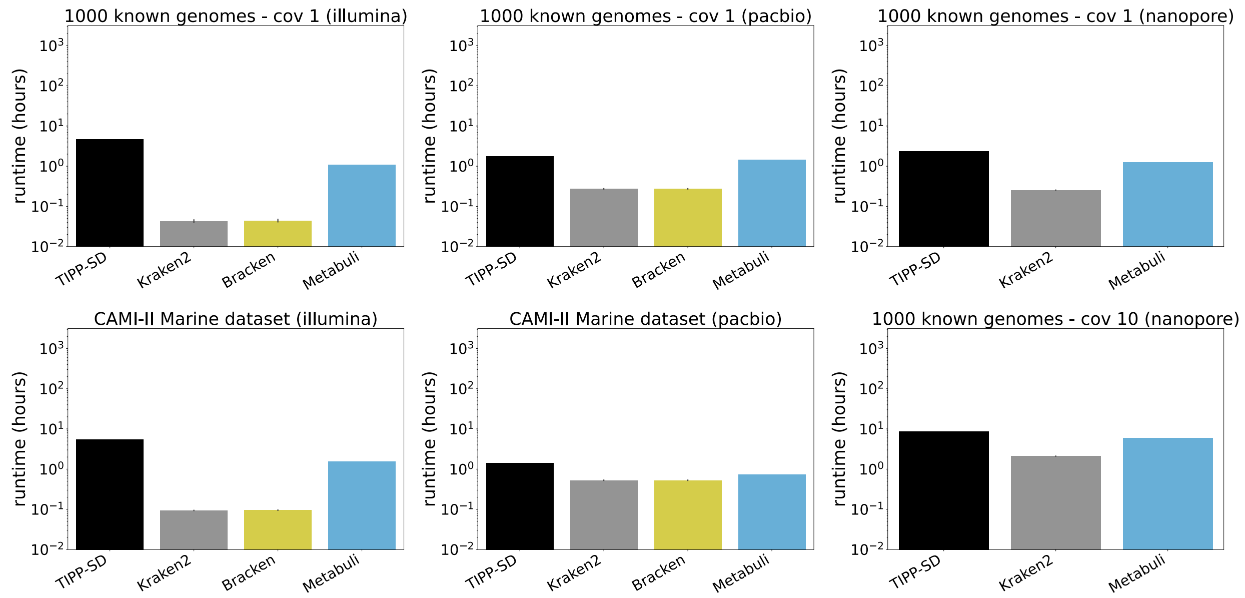

Fig I: **Experiment 2: Runtime in hours (in log-scale) of TIPP-SD, Kraken2, Bracken, and Metabuli.** TIPP-SD is run in default mode, Kraken2, Bracken, and Metabuli use all reads; Kraken2 and Bracken are based on confidence scores, and Metabuli is based on read count. For Kraken2 and Bracken, the average and standard error runtime are based on 50 different tested confidence scores. Bracken was not run for nanopore reads due to computational limitations for building the database with long reads.

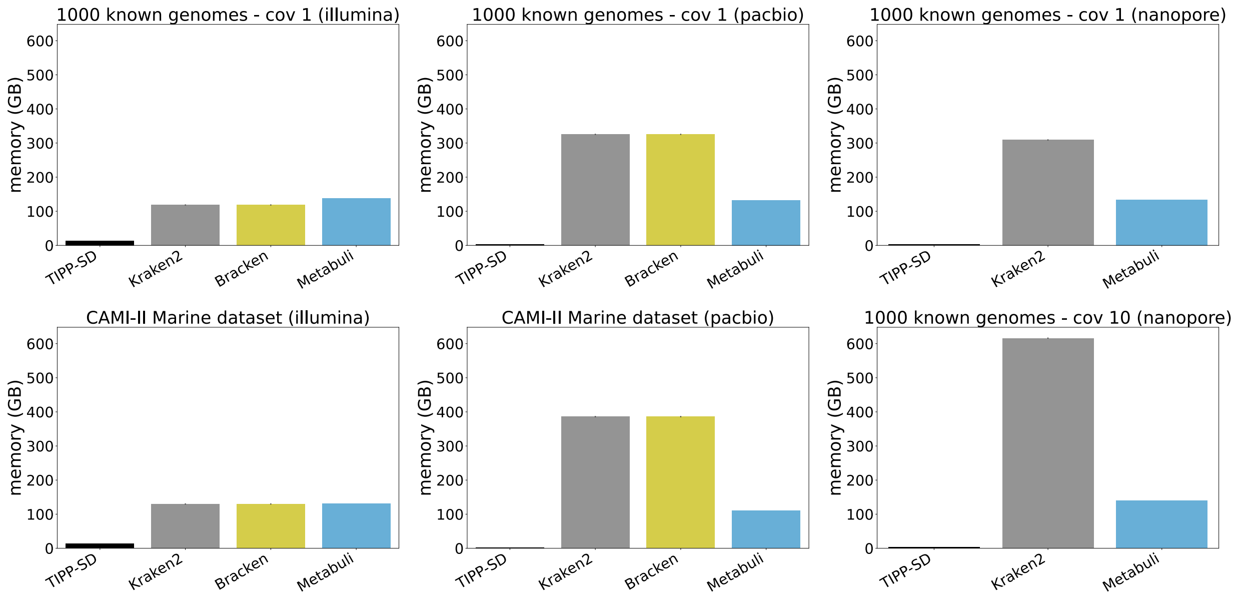

Fig J: **Experiment 2: Memory usage in GBs of TIPP-SD, Kraken2, Bracken, and Metabuli.** All methods other than TIPP-SD use all reads (unfiltered). For Kraken2 and Bracken, the runtime for each confidence score is averaged, and the standard error is shown. Bracken was not run for nanopore reads due to computational limitations for building the database with long reads.

Table C: **Experiment 2: Runtime breakdown on alignment and placement in hours for TIPP-SD.**

Dataset	Alignment (h)	Placement (h)
1000 known genomes - cov 1 (illumina)	3.0	1.8
1000 known genomes - cov 1 (pacbio)	0.5	1.3
1000 known genomes - cov 1 (nanopore)	0.8	1.6
1000 known genomes - cov 10 (nanopore)	4.2	4.3
CAMI-II Marine dataset (illumina)	3.4	2.1
CAMI-II Marine dataset (pacbio)	0.6	0.8

B.3 Additional results for Experiment 3

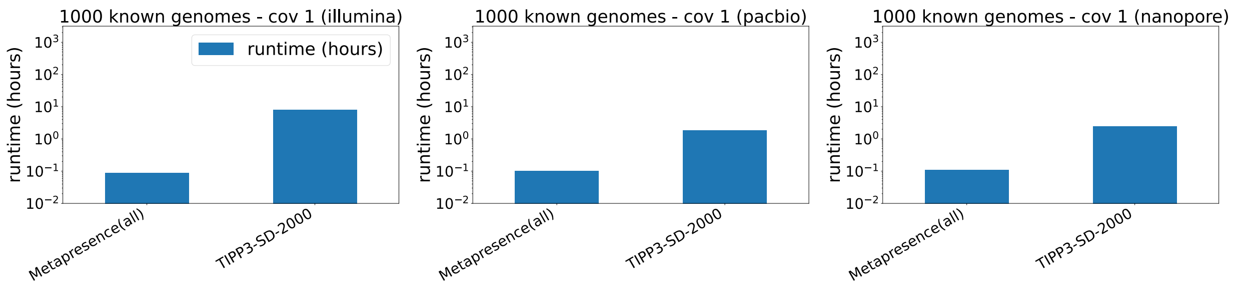

(a) Runtime in hours (log-scale)

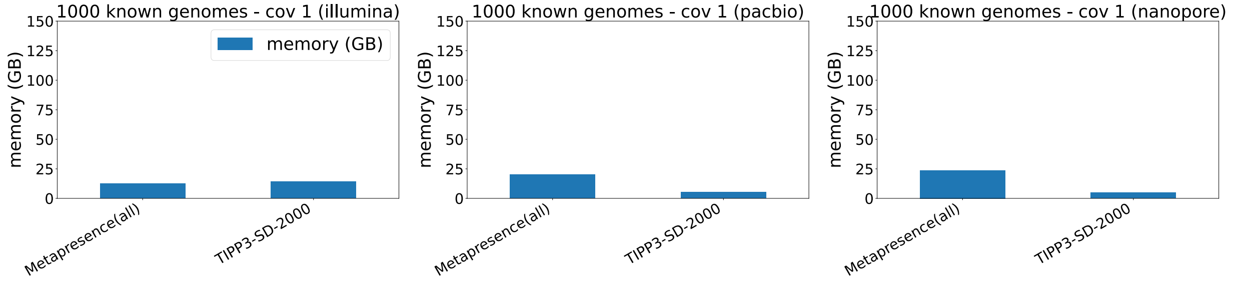

(b) Memory in GBs

Fig K: **Experiment 3: Runtime and memory usage of TIPP-SD-2000 and Metapresence on 1000 known genomes datasets**. Analyses are shown for Illumina, PacBio, and nanopore reads. Metapresence aligns reads with Bowtie2 for Illumina and Minimap2 for PacBio and nanopore reads. Coverages for the 1000 species datasets are 1.

Table D: **Experiment 3: AUPR for precision and recall curves of TIPP-SD-2000 and Metapresence.**

Queryset	1000 Genomes Illumina (Cov1)	1000 Genomes PacBio (Cov1)	1000 Genomes Nanopore (Cov1)
TIPP-SD-2000	0.992	0.995	0.986
Metapresence(all)	0.080	0.917	0.886

References

[1] P. Danecek, J. K. Bonfield, J. Liddle, J. Marshall, V. Ohan, M. O. Pollard, A. Whitwham, T. Keane, S. A. McCarthy, R. M. Davies, and H. Li. Twelve years of SAMtools and BCFtools. *GigaScience*, 10(2):giab008, 02 2021.

[2] C. Jain, L. M. Rodriguez-R, A. M. Phillippy, K. T. Konstantinidis, and S. Aluru. High throughput ANI analysis of 90K prokaryotic genomes reveals clear species boundaries. *Nat Commun*, 9(1):5114, Nov. 2018.

[3] B. Langmead, C. Wilks, V. Antonescu, and R. Charles. Scaling read aligners to hundreds of threads on general-purpose processors. *Bioinformatics*, 35(3):421–432, 02 2019.

[4] H. Li. Minimap2: pairwise alignment for nucleotide sequences. *Bioinformatics*, 34(18):3094–3100, Sept. 2018.

[5] J. Lu, N. Rincon, D. E. Wood, F. P. Breitwieser, C. Pockrandt, B. Langmead, S. L. Salzberg, and M. Steinegger. Metagenome analysis using the Kraken software suite. *Nature protocols*, 17(12):2815–2839, 2022.

[6] C. Shen, E. Wedell, M. Pop, and T. Warnow. TIPP3 and TIPP3-fast: Improved abundance profiling in metagenomics. *PLOS Computational Biology*, 21(4):e1012593, Apr. 2025. Publisher: Public Library of Science.

[7] E. Wedell, C. Shen, and T. Warnow. BSCAMPP: Batch-Scaled Phylogenetic Placement on Large Trees. *IEEE/ACM Trans. Comp. Biol. Bioinf.*, 22(4):1593–1605, 2025. doi: 10.1109/TCBBIO.2025.3562281.
